# Case Report: GATA2 deficiency in two families with novel frameshift variants highlighting phenotypic diversity and need for early diagnosis

**DOI:** 10.3389/fimmu.2025.1644552

**Published:** 2025-07-31

**Authors:** Miko Morimoto, Takuro Nishikawa, Atsushi Hijikata, Hiroshi Kasabata, Nobuhisa Maeda, Shuji Kanmura, Shogo Horikawa, Jun Nagahama, Aki Nakamura, Tatsuro Nakamura, Takanari Abematsu, Shunsuke Nakagawa, Kazuhiro Shimura, Satoshi Narumi, Hirokazu Kanegane, Yasuhiro Okamoto

**Affiliations:** ^1^ Department of Pediatrics, Graduate School of Medical and Dental Sciences, Kagoshima University, Kagoshima, Japan; ^2^ School of Life Sciences, Tokyo University of Pharmacy and Life Sciences, Tokyo, Japan; ^3^ Department of Clinical Laboratory Medicine, Kagoshima University Hospital, Kagoshima, Japan; ^4^ Digestive and Lifestyle Diseases, Kagoshima University Graduate School of Medical and Dental Sciences, Kagoshima, Japan; ^5^ Department of Pediatrics, Keio University School of Medicine, Tokyo, Japan; ^6^ Department of Child Health and Development, Graduate School of Medical and Dental Sciences, Institute of Science Tokyo, Tokyo, Japan

**Keywords:** acute myeloid leukemia, Crohn disease, GATA2 gene, hematopoietic stem cell transplantation, myelodysplastic syndrome, structural analysis

## Abstract

**Background:**

GATA2 deficiency, a syndrome caused by heterozygous loss-of-function variants in the *GATA2* gene, is characterized by immunodeficiency, bone marrow failure, and predisposition to myeloid neoplasms. Its clinical presentation is highly variable, making early diagnosis challenging. Although GATA2 deficiency has been linked to systemic inflammation, gastrointestinal involvement mimicking inflammatory bowel disease (IBD) is extremely rare.

**Case presentation:**

This report presented the case of two adolescent boys with no family history of novel heterozygous frameshift *GATA2* variants. Notably, Patient 1 initially presented with clinical and endoscopic features strongly suggestive of Crohn’s disease, including weight loss, perianal abscess, and characteristic intestinal ulcers, before developing acute myeloid leukemia with monosomy 7. This is a rare presentation of GATA2 deficiency manifesting initially with Crohn’s disease-like symptoms. Patient 2 presented with intractable cutaneous warts and pancytopenia, later diagnosed as myelodysplastic syndrome with der(1;7)(q10;p10). Both patients harbored novel *GATA2* frameshift variants predicted to eliminate the DNA-binding domain, suggesting a loss-of-function mechanism.

**Conclusion:**

These cases expand the phenotypic spectrum of GATA2 deficiency and highlight that atypical IBD-like symptoms, including Crohn’s disease-like presentations, may cause an initial manifestation. GATA2 deficiency should be considered in patients with IBD-like symptoms, refractory skin disorders, and hematological abnormalities. Early genetic testing and family screening are essential to ensuring timely diagnosis and curative hematopoietic stem cell transplantation before progression to advanced myeloid disease.

## 1 Introduction

GATA2 deficiency, caused by heterozygous loss-of-function variants in the *GATA2* gene, results in haploinsufficiency and manifests as a broad spectrum of clinical features. These include susceptibility to mycobacterial infections and viral infections, bone marrow failure, myeloid neoplasms, including familial myelodysplastic syndrome (MDS) and acute myeloid leukemia (AML), sensorineural hearing loss, and lymphedema (1, 2). GATA2 deficiency is also associated with a decrease or complete loss of B cells, monocytes, natural killer (NK) cells, and dendritic cells. Although the severity of immunodeficiency varies, infections caused by human papillomavirus, herpesviruses, and atypical mycobacteria are particularly characteristic of this condition (3–5). The incidence of myeloid tumors initially occur at about 15 years of age, with approximately 80% of cases diagnosed by the age of 40 (3). GATA2 deficiency has a highly variable penetrance, and infections or other factors may affect epigenetic mechanisms that modulate disease onset and severity (3).

Over 100 *GATA2* variants have been identified, spanning both coding and noncoding regions (6). These pathogenic variants have been grouped into three categories: null variants (approximately 60%), missense variants, and regulatory or intronic variants (2, 7). Frameshift variants are the most common subtype among the null variants and tend to localize upstream of the zinc finger domains (2, 7). Genotype–phenotype associations in GATA2 deficiency remains poorly understood (2, 6–8), and intrafamilial phenotypic variability has been reported (9).

Monogenic inflammatory bowel disease (IBD) is a rare genetically driven form of IBD that typically presents in early childhood, often before 6 years of age (10). Unlike the more common polygenic forms of IBD, such as Crohn’s disease and ulcerative colitis, which are influenced by environmental factors, monogenic IBD arises from variants in a single gene. Although extremely rare, cases of GATA2 deficiency with Crohn’s-like intestinal involvement have been reported (11); however, detailed clinical characterization remains limited.

This report presents two cases of GATA2 deficiency with novel frameshift variants. One patient initially presented with Crohn’s disease, and another patient initially presented mainly with skin symptoms, revealing the phenotypic diversity of GATA2 deficiency. These novel variants appear to result in a truncated variant in which the DNA-binding domain and beyond are missing.

## 2 Case description

This study was conducted in accordance with the principles of the Declaration of Helsinki and was approved by the Ethics Committee on Clinical Research of the Sakuragaoka Campus, Kagoshima University (No. 250053). Informed consent was obtained from the patient and their parents for participation in this study and publication of the data and images.

**Figure 1:**
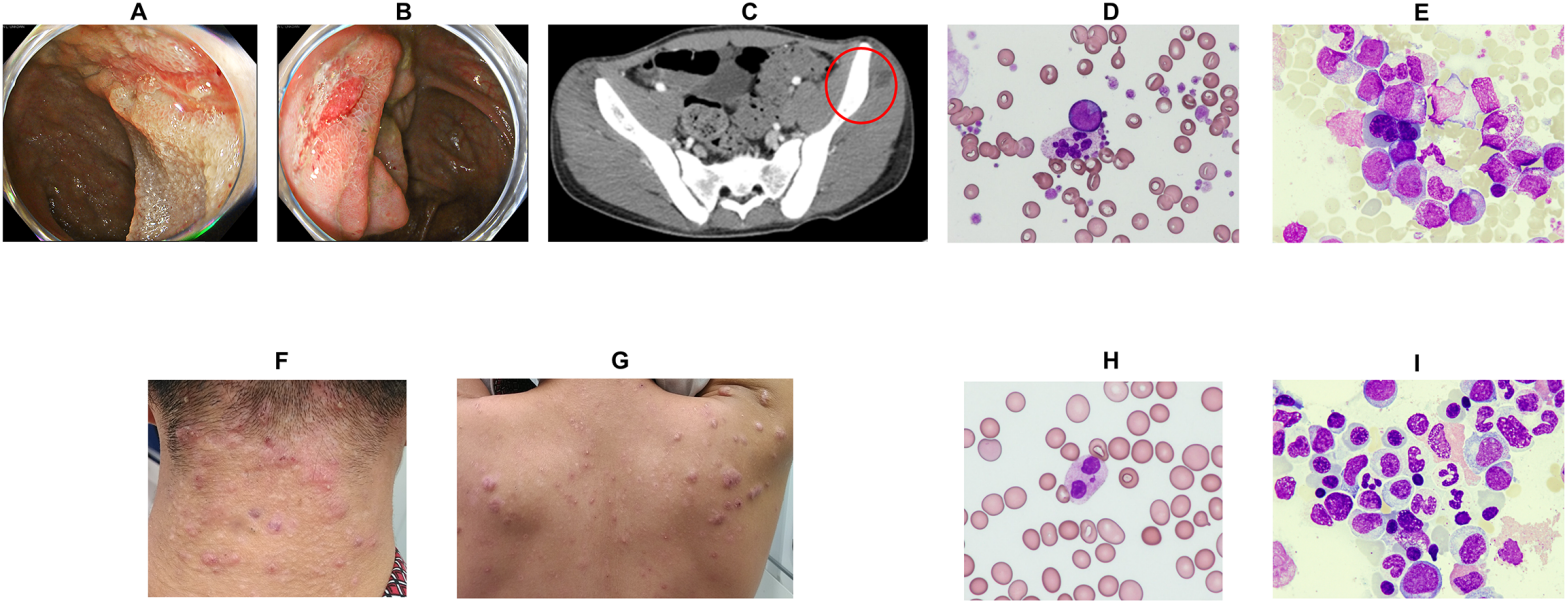
Clinical symptoms of patients and peripheral blood/bone marrow examination findings. **(A)** Lower endoscopy revealed longitudinal ulcers at the terminal ileum and discontinuous aphthous-like erosions from the cecum to the rectum. **(B)** Lower endoscopy revealed an ulcer at the Bauhin valve. **(C)** Pelvic contrast-enhanced computed tomography revealed a subcutaneous abscess in the left buttock (red circle). **(D)** Peripheral blood tests showed hypergranular neutrophils, giant platelets, and abnormal cells (Wright**–**Giemsa stain ×400). **(E)** Bone marrow examination revealed an increase in abnormal cells (Wright**–**Giemsa stain ×400). Refractory warts were noted in the posterior neck **(F)** and back **(G)**. **(H)** Peripheral blood examination revealed hypergranular neutrophils (Wright**–**Giemsa stain ×400). **(I)** Bone marrow examination revealed multilineage dysplasia. (Wright**–**Giemsa stain ×400).

Patient 1 was a 16-year-old male referred to a general hospital 6 months prior with a history of recurrent stomatitis and subcutaneous abscesses. The patient’s past medical history, growth, and development were unremarkable. The patient’s mother had a history of ulcerative colitis. Despite receiving antimicrobial treatment, his symptoms recurred. Peripheral blood tests revealed morphological abnormalities, such as hypersegmented neutrophils and giant platelets, although blood cell counts were within normal limits. Three months before admission, he was referred to a gastroenterology hospital owing to fever, loss of appetite, weight loss of approximately 10 kg, and occult blood in the stool. Endoscopy revealed longitudinal ulcers at the terminal ileum, malformed ulcers on the ileocecal valve, and multiple longitudinal ulcers and aphthae in the small intestine (**Figures 1A, B**). Pathology showed submucosal inflammatory cell infiltration without epithelial granulomas. Endoscopic findings of multiple longitudinal ulcers and anorectal lesions supported the diagnosis of Crohn’s disease affecting the small and large bowel. Treatment was initiated with elemental nutrition and 5-aminosalicylic acid, but persistent fever and anorexia prompted prednisolone (30 mg/day) and infliximab, resulting in symptom improvement. However, the patient developed a perianal abscess and recurrent fever (**Figure 1C**). He was treated with antimicrobials, but the fever persisted. He also developed limb pain and abnormal peripheral blood cell findings, raising suspicion for a hematological disorder and was, therefore, referred to our department for further evaluation and admission.

Physical examination revealed a height of 171 cm, weight of 59.0 kg, temperature of 37.7°C, heart rate of 107 beats/min, and blood pressure of 113/60 mmHg. No stomatitis or lymphadenopathy was observed, and both the heart and respiratory sounds were normal. Notable findings included extremity pain, a pyogenic powdery mass in the buttocks, and perianal abscess. Initial laboratory tests (**Table 1**) revealed leukocytosis (white blood cell [WBC] count: 11,080/μL, with 28.5% blasts, 37% granulocytes, 19% lymphocytes (2,105/μL), and dysplastic neutrophils. Anemia and thrombocytopenia with giant platelets were also observed (**Figure 1D**). Serum immunoglobulin (Ig) G, IgA, and IgM levels were normal (1,568, 126, and 142 mg/dL, respectively; normal ranges: 830–1,740, 90–400, and 31–200 mg/dL, respectively). Lymphocyte subset analysis revealed reduced CD3^+^ T cells (48.8%; normal range: 54.3–81.9%) and markedly decreased CD19^+^ B cells (0.4%; normal: 2.9–20.1%).

**Table 1:** Laboratory findings at the initial presentation.

Laboratory data			
	Patient 1	Patient 2	Normal value
WBC (/μL)	11,080	2,150	4,400–9,100
Neutrophil (%)	37	56	30–40
Pelger–Huët anomaly	+	+	–
Blast (%)	28.5	0	–
Monocyte (%)	15.5	0	4.3–10
Lymphocyte (/μL)	2,105	839	1050–2880
CD3^+^ T cells (%)	48.8	95	58–84
CD4^+^ T cells (% of CD3^+^)	NA	51	25–56
CD8^+^ T cells (% of CD3^+^)	NA	40	17–44
CD19^+^ B cells (%)	0.4	3	5-24
IgD^-^CD27^+^ memory B cells (% of CD19^+^)	NA	19.4	0.9–21.2
CD16^+^CD56^+^ NK cells (%)	NA	1	5–38
IgG (mg/dL)	1,568	1,286	870–1,700
IgA (mg/dL)	126	130	110–410
IgM (mg/dL)	142	157	33–190

NA, not available data; NK, natural killer.

Higher-than-reference values are highlighted in red and lower-than-reference values are highlighted in blue.

A diagnosis of AML-related myelodysplasia was established based on a bone marrow (BM) smear, which showed 28.1% myeloblasts of the M1 subtype according to the French-American-British classification (**Figure 1E**). Flow cytometry demonstrated that the blasts expressed CD13, CD33, CD34, CD38, CD71, CD117, and myeloperoxidase. Cytogenetic analysis of BM cells revealed a karyotype of 45,XY,-7 (19/20 cells). Additional findings included hypoalbuminaemia (Alb 2.5 g/dL) and elevated inflammatory markers (C-reactive protein [CRP]: 21.0 mg/dL, ferritin: 2,138 ng/mL). Follow-up lower gastrointestinal endoscopy showed resolution of the previously observed longitudinal ulcers and aphthae. The gastrointestinal findings were interpreted as Crohn’s disease-like lesions, although atypical for Crohn’s disease.

Patient 2 was a 17-year-old male referred to a general hospital with persistent fatigue and intractable skin lesions. The patient’s past medical history, growth, and development were unremarkable. The patient’s mother had a history of breast cancer and died of a brain hemorrhage at 43-years-old. Peripheral blood examination revealed abnormal blood cell morphology, such as hypersegmented neutrophils, giant platelets, as well as pancytopenia. He was subsequently referred to our hospital for further evaluation.

Physical examination revealed a height of 162 cm, weight of 51.6 kg, temperature of 36.8°C, heart rate of 100 beats/min, and blood pressure of 121/72 mmHg. No stomatitis or lymphadenopathy was observed, and both heart and respiratory sounds were normal. Numerous warts were observed on the skin, mainly on the neck and trunk (**Figures 1F, G**). Initial laboratory tests (**Table 1**) revealed leukocytopenia (WBC count: 2,150/μL, 56% granulocytes, 39% lymphocytes (839/μL) and dysplastic neutrophils (**Figure 1H**). Anemia and thrombocytopenia with giant platelets were also present. Serum IgG, IgA, and IgM levels were normal (1,286, 130, and 157 mg/dL, respectively; normal ranges: 830–1,740, 90–400, and 31–200 mg/dL, respectively). Lymphocyte subset analysis showed that CD3^+^ T cells comprised 95% (normal range: 50–83%). NK cells comprised 1% (normal: 5–41%), CD19^+^ B cells comprised 3% (6–23%), and dendritic cells were absent. A diagnosis of MDS with low blasts was made based on a BM smear that showed 1.0% myeloblasts (**Figure 1I**). Cytogenetic analysis revealed a karyotype of 48,XY,+1,der(1;7) (q10;p10),+8,+21 [14/20 cells].

### 2.1 Genetic analysis

**Figure 2:**
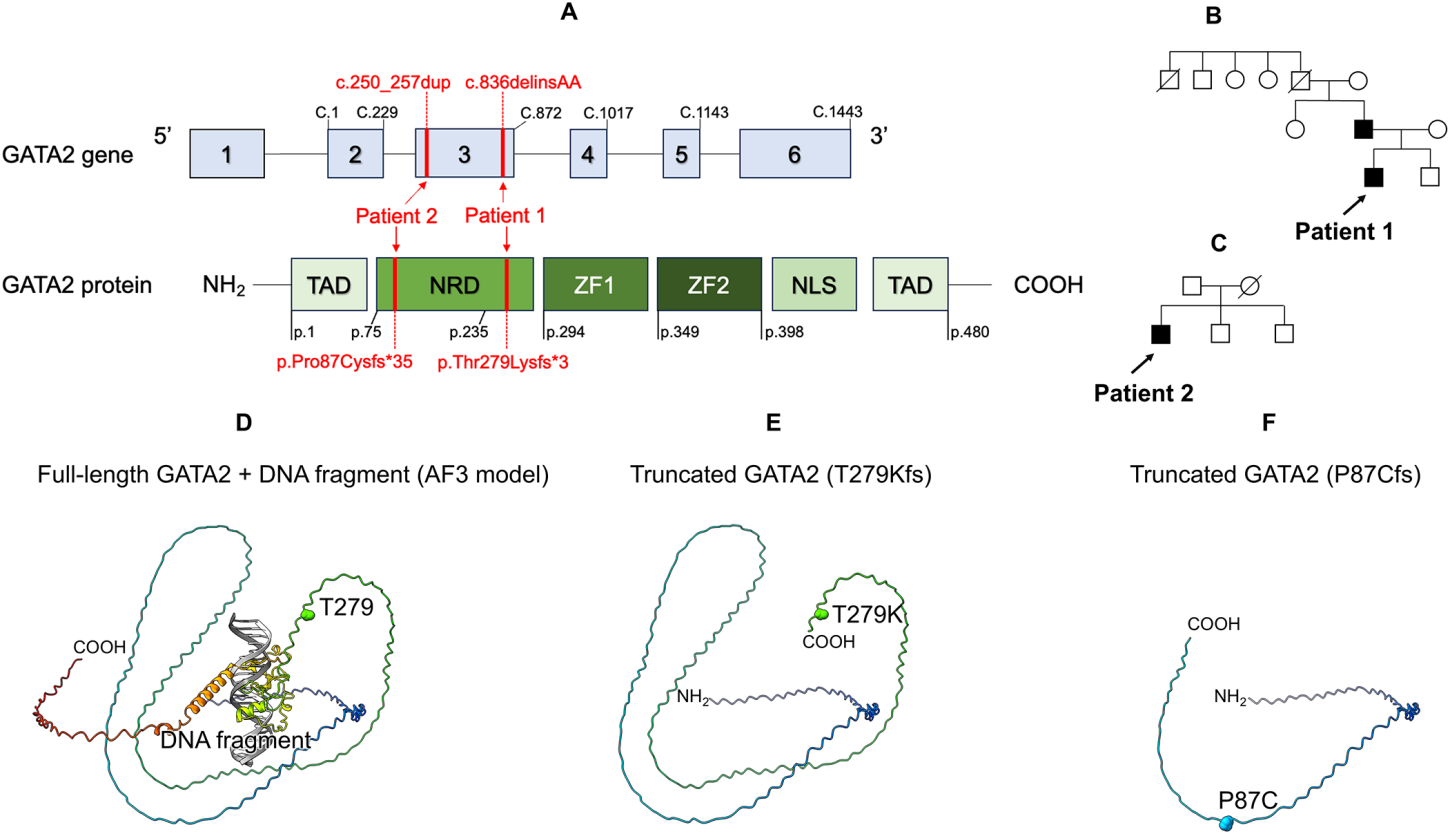
Genetic, protein structure analysis, and family tree. **(A)** Location of the *GATA2* variant in both patients. Schematic representation of the three domains of the GATA2 protein and six exons of GATA2. **(B)** Family tree of patient 1. **(C)** Family tree of patient 2. **(D)** Structural analysis of GATA2 protein. Full-length GATA2 + DNA fragment (AF3 model) **(E)** Truncated GATA2 (T279Kfs). **(F)** Truncated GATA2 (P87Cfs). TAD, N-terminal Transactivation Domain; NRD, N-terminal Regulatory Domain; ZF1, C-terminal zinc finger; ZF2, N-terminal zinc finger; NLS, Nuclear Localization Signal.

In Patient 1, targeted panel sequencing of genes associated with disorders of sex development and gonadal/adrenal dysfunction (*AAAS, AR, CDKN1C, CHD7, CPOX, CYP11A1, CYP17A1, DHX37, DMRT1, GATA2, HSD17B3, HSD3B2, LGR4, MAMLD1, NR5A1, NNT, NR0B1, POLE, RSPO1, SAMD9, SOX9, SRD5A2, SRY, STAR, WNT4*, and *WT1)*, including the *SAMD9* gene, was performed due to initial suspicion of as MIRAGE syndrome (12). We identified a novel heterozygous frameshift variant (NM_032638.5:c.836delinsAA, p.Thr279Lysfs*3) of *GATA2* (**Figure 2A**). Family screening (father, mother, and younger brother) using genomic DNA from peripheral blood cells revealed the same *GATA2* variant in the father (**Figure 2B**). The patient’s father is 56-years-old and has GATA2 deficiency, but has remained asymptomatic to date. The paternal grandfather died of hypertrophic cardiomyopathy at 69 years of age.

In Patient 2, targeted panel sequencing of familial dendritic cell deficiency (*GATA2, CSF2RA, CSF2RB, IRF7*, and *IRF8)* was performed using genomic DNA from buccal mucosa swabs. We identified a novel heterozygous frameshift variant (NM_032638.5:c.250_257dup, p.Pro87Cysfs*35) of *GATA2* (**Figure 2A**). Family screening revealed that neither the father or patient’s brothers carried the same *GATA2* variant (**Figure 2C**).

### 2.2 Protein structure analysis

The model structure of the human GATA2 complex and a DNA fragment were constructed using the AlphaFold server (https://alphafoldserver.com), running AlphaFold 3 (13). The amino acid sequence of human GATA2 was retrieved from the UniProt database (accession number: P23769), and the DNA sequence was derived from the crystal structure of the GATA3-DNA complex (PDB code: 4HCA). Both novel variants are truncated variants in which the DNA-binding domain and beyond are missing and are considered loss-of-function variants (**Figures 2D–F**).

### 2.3 Flowcytometry

Peripheral blood was used as the sample material. Whole blood (50 µL) was added to tubes for analysis. Flow cytometry was performed using a BD FACS Canto II (BD Biosciences), and data were analyzed with BD FACSDiva software (BD Biosciences). Unless otherwise stated, the following antibodies were obtained from BD Biosciences: antigen–fluorochrome (clone); CD14 FITC (M5E2), CD16 FITC (3G8), CD19 (HIB19), CD3 FITC (UCHT1; Beckman Coulter), CD11c PE (B-ly6), CD45 PerCP (2D1), CD123 APC (7G3), HLA-DR BV421 (G46-6), and CD56 BV510 (NCAM16.2). After staining (20 minutes at room temperature in the dark), red blood cells were lysed using BD FACS™ Lysing Solution, followed by washing with BD™ CellWASH. Flow cytometric analysis was then performed. Lin^−^ cells (negative for CD14, CD16, CD19, and CD3) were gated, and CD56^+^ HLA-DR^−^ cells were excluded from this population. Dendritic cells were subsequently classified into HLA-DR^+^ CD11c^+^ (myeloid DCs) and HLA-DR^+^ CD123^+^ (plasmacytoid DCs) subsets.

### 2.4 Patient’s treatment course and follow-up

**Figure 3:**
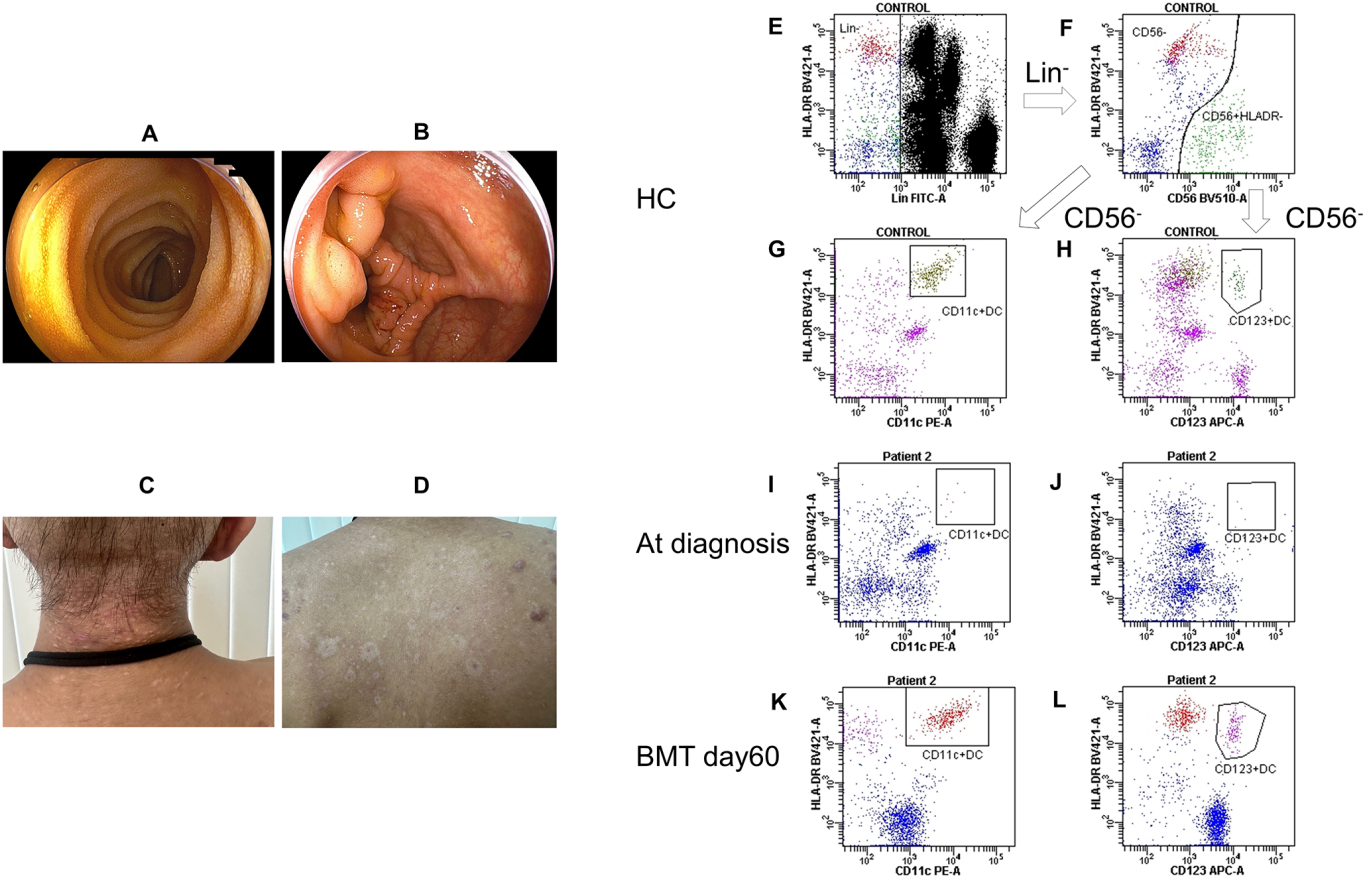
Post-treatment patient findings and changes in dendritic cell counts. **(A, B)** Lower endoscopy before peripheral blood cell transplantation showed improvement and disappearance of longitudinal ulcers and aphthous-like erosions. **(C, D)** Skin lesions after bone marrow transplantation improved immediately. **(E)** Lin^−^ cells (negative for CD14, CD16, CD19, and CD3) were gated in healthy control. **(F)** CD56^+^ HLA-DR^−^ cells were excluded in healthy control. **(G)** The square contains CD11c/HLA-DR-positive cells in healthy control. **(H)** The square contains CD123/HLA-DR-positive cells in healthy control. **(I)** The square contains CD11c/HLA-DR-positive cells at the time of diagnosis of Patient 2. **(J)** The square contains CD123/HLA-DR-positive cells at the time of diagnosis in Patient 2. **(K)** The square contains CD11c/HLA-DR-positive cells on day 60 post-BMT in Patient 2. **(L)** The square contains CD123/HLA-DR-positive cells on day 60 post-BMT in Patient 2. BMT, bone marrow transplantation; HC, healthy control; Lin, lineage.

Patient 1 received three cycles of chemotherapy and achieved complete remission. The patient underwent human leukocyte antigen (HLA)-haploidentical peripheral blood stem cell transplantation (PBSCT) from his brother (non-carrier), using post-transplantation cyclophosphamide. The conditioning regimen comprised fludarabine (120 mg/m^2^) and melphalan (180 mg/m^2^). Four months following the PBSCT, the patient relapsed. Chemotherapy was administered again but did not achieve remission, subsequently, the patient underwent bone marrow transplantation (BMT) from an HLA-matched unrelated donor. The conditioning regimen comprised busulfan (targeted total area under the concentration-time curve [AUC] 20,000 μM×min) and melphalan (180 mg/m^2^). However, 10 months after the BMT, the patient died of AML. No treatment for Crohn’s disease was administered at our hospital; however, there was no recurrence of symptoms. Moreover, endoscopic findings pre-PBSCT showed further improvement, with normal mucosal appearance (**Figures 3A, B**).

Patient 2 underwent BMT from an HLA-matched brother 2 months after diagnosis. A BM examination was conducted immediately before transplantation, revealing an increase in blasts to 7.8%. No specific treatment was administered for the skin lesions before hematopoietic stem cell transplantation (HSCT); instead, hygiene was maintained with the expectation of improvement following transplantation. The conditioning regimen included busulfan (targeted total AUC: 20,000 μM×min) and cyclophosphamide (120 mg/kg). Three months after BMT, the patient remained in remission without evidence of graft-*vs*-host disease. Notably, his skin lesions improved rapidly after transplantation (**Figures 3C, D**). At diagnosis, dendritic cells (CD11c/HLA-DR-positive and CD123/HLA-DR-positive) were scarcely present compared with that of a healthy control (**Figures 3E–J**). Following BMT, a substantial number of donor-derived dendritic cells became detectable (**Figures 3K, L**).

## 3 Discussion

*GATA2* frameshift variants represent a significant subset of pathogenic variants in GATA2 deficiency, accounting for approximately 23% of all cases. Among null variants, they are the most common subtype (2, 7). These variants typically occur in exons 3–5 upstream of the zinc finger domains and introduce premature stop codons. Consequently, they result in either truncated, non-functional proteins or lead to transcript degradation via nonsense-mediated mRNA decay, culminating in haploinsufficiency. The two variants identified in our patients were located upstream of the zinc-finger domains and are presumed to result in haploinsufficiency. Frameshift variants in *GATA2* are often associated with early-onset clinical manifestations, typically appearing in childhood or adolescence. In a French-Belgian cohort (2), patients with frameshift or other null variants presented with symptoms at a median age of 13 years, often with recurrent bacterial infections, such as pneumonia or cellulitis. Unlike missense variants, associated with a primary diagnosis of myeloid malignancy, frameshift variants tend to manifest initially as immunodeficiency or cytopenia, preceding leukemic transformation. Consistent with these findings, our two patients presented with immunodeficiency symptoms during adolescence, before progressing to leukemia. In this case, only structural analysis of the protein was conducted, and functional characterization was not performed. However, the identification of a frameshift variant well upstream of the zinc finger domain strongly suggests a significant impact on protein function, supporting its pathogenic potential.

According to a report by the European Working Group of MDS in Childhood, germline *GATA2* variants account for 7% of all primary MDS cases (14). Variant carriers were older at diagnosis and more likely to present with monosomy 7 and advanced disease compared with that of MDS cases lacking *GATA2* variants. Among the abnormal karyotypes observed, monosomy 7 was most common (68%), followed by trisomy 8 (9%), and then unbalanced aberration der(1;7) in 7% of patients (14). The der(1;7)(q10;p10) karyotype observed in Patient 2 is rare, reported in only 0.8% of pediatric MDS cases. However, 73% of these cases have been reported to have a germline *GATA2* variant (15). According to previous reports (15, 16), MDS with der(1;7)(q10;p10) generally has a poor prognosis, although not as poor as cases with monosomy 7 (16, 17). Therefore, in pediatric adolescent young adult cases of MDS or AML with monosomy 7 or der(1;7)(q10;p10), it is essential to perform germline *GATA2* testing. In cases of GATA2 deficiency associated with MDS/AML, HSCT is typically indicated. It is recommended that family screening be conducted early, particularly when considering a related. Family screening should be offered to all first-degree relatives, as identification of asymptomatic GATA2-deficient patients could allow the exclusion of potential HSCT donors and investigation of risk factors that may explain the phenotypic differences. Furthermore, genetic panel testing for hematologic malignancies is expected to become routine practice and rapidly adopted worldwide. Although this test primarily examines somatic changes in leukemia cells, it also identifies changes in the germline, leading to an expected improvement in the diagnosis of GATA2 deficiency. The reason Patient 1’s father has remained asymptomatic may involve not only the genetic variant itself but also epigenetic or other modifier factors. Differences in DNA methylation and allele-specific expression have been reported between symptomatic and asymptomatic individuals, suggesting a role for these factors in disease penetrance (18). Patient 2’s mother had breast cancer and also suffered a cerebral hemorrhage, leading to her death at the young age of 43. Given that GATA2 deficiency is inherited in an autosomal dominant manner, and that Patient 2’s father did not carry the GATA2 variant, it is possible—although genetic testing was not performed—that the mother may have had GATA2 deficiency. HSCT should be considered in patients with immunodeficiency without myeloid neoplasms, as performing HSCT before patients develop malignancies or severe or recurrent infections that cause organ failure is likely to increase their long-term survival.

GATA2 deficiency presents a broad phenotypic spectrum; recognizing its clinical features is essential for early genetic diagnosis, appropriate management, preventive care, and family screening. Clinical presentations that should prompt genetic screening for GATA2 deficiency include idiopathic cytopenia—affecting neutrophils, monocytes, dendritic, B, and NK cells—idiopathic CD4 lymphocytopenia, MDS, aplastic anemia in adolescents and young adults, pulmonary alveolar proteinosis, congenital lymphedema, and marked susceptibility to human papillomaviruses and nontuberculous mycobacteria, among others (3, 14). Patient 1 was initially diagnosed with Crohn’s disease and received treatment. At initial presentation, MIRAGE syndrome was suspected based on the presence of AML with monosomy 7, gastrointestinal abnormalities, and infection susceptibility. Consequently, a targeted gene panel analysis including *SAMD9* and *GATA2* was performed, leading to a diagnosis of GATA2 deficiency. GATA2 deficiency is believed to cause systemic inflammation involving multiple organs (19). Our case provides additional clinical and endoscopic evidence supporting a potential association with monogenic IBD. Several studies have shown that dendritic cell dysfunction—such as reduced dendritic cell subsets, aberrant cytokine signaling, and impaired antigen presentation—can contribute to Crohn’s disease pathogenesis (20, 21). Crohn-like colitis has also been reported in other primary immunodeficiencies involving dendritic cell impairment, such as Wiskott-Aldrich syndrome (22). These findings suggest that defective dendritic cell-mediated immune regulation may broadly underlie the development of intestinal inflammation in GATA2 deficiency and related conditions. However, the association between GATA2 deficiency and Crohn’s-like colitis is based on a single case. As only panel-based testing was performed, we cannot exclude the possibility of other genetic contributors, such as NOD2 variants or other monogenic IBD-associated variants. Patient 2 was suspected of GATA2 deficiency based on skin symptoms suggestive of human papillomavirus infection and monocyte/dendritic cell deficiency. HSCT led to the rapid resolution of skin lesions. Dendritic cells are not routinely tested; however, in cases where GATA2 deficiency is suspected, it is recommended to actively perform CD11c, CD123, and HLA-DR testing. Cells expressing CD11c and HLA-DR are classified as myeloid dendritic cells, whereas those expressing CD123 and HLA-DR are classified as plasmacytoid dendritic cells (23). In Patient 2, family genetic screening was completed, and BMT was performed within 2 months of the initial visit, shortly before leukemia onset. This intervention was possible because GATA2 deficiency was considered from the beginning of the clinical evaluation. In contrast, Patient 1 was diagnosed with GATA2 deficiency >6 months after initiating leukemia treatment, as the atypical initial presentation with Crohn’s disease delayed recognition. Moving forward, it may be necessary to consider GATA2 deficiency in patients with MDS or cytopenia presenting with IBD. One limitation of this study is the lack of functional validation of the identified GATA2 variants. Although pathogenicity was inferred based on the location of the frameshift variants and structural predictions using protein modeling, transcript-level analyses were not performed. The absence of these direct functional assays prevents definitive conclusions regarding their biological impact.

## 4 Conclusions

We report two families with novel frameshift variants in *GATA2* that were incidentally diagnosed with myeloid neoplasms. One patient presented with Crohn’s disease, a very rare manifestation of GATA2 deficiency. Myeloid neoplasms with episodes suggestive of immunodeficiency/IBD and a marked reduction or absence of B cells, monocytes, NK cells, and dendritic cells should be actively screened for *GATA2* variants.

## Data Availability

The datasets for this article are not publicly available due to concerns regarding participant/patient anonymity. Requests to access the datasets should be directed to the corresponding author.
